# The epidemiological impact of antiretroviral use predicted by mathematical models: a review

**DOI:** 10.1186/1742-7622-2-9

**Published:** 2005-09-10

**Authors:** Rebecca F Baggaley, Neil M Ferguson, Geoff P Garnett

**Affiliations:** 1 Department of Infectious Disease Epidemiology, Imperial College London, Norfolk Place, London W2 1PG, UK

## Abstract

This review summarises theoretical studies attempting to assess the population impact of antiretroviral therapy (ART) use on mortality and HIV incidence. We describe the key parameters that determine the impact of therapy, and argue that mathematical models of disease transmission are the natural framework within which to explore the interaction between antiviral use and the dynamics of an HIV epidemic. Our review focuses on the potential effects of ART in resource-poor settings. We discuss choice of model type and structure, the potential for risk behaviour change following widespread introduction of ART, the importance of the stage of HIV infection at which treatment is initiated, and the potential for spread of drug resistance. These issues are illustrated with results from models of HIV transmission. We demonstrate that HIV transmission models predicting the impact of ART use should incorporate a realistic progression through stages of HIV infection in order to capture the effect of the timing of treatment initiation on disease spread. The realism of existing models falls short of properly reproducing patterns of diagnosis timing, incorporating heterogeneity in sexual behaviour, and describing the evolution and transmission of drug resistance. The uncertainty surrounding certain effects of ART, such as changes in sexual behaviour and transmission of ART-resistant HIV strains, demands exploration of best and worst case scenarios in modelling, but this must be complemented by surveillance and behavioural surveys to quantify such effects in settings where ART is implemented.

## Introduction

The epidemiological impact of widescale use of (highly active) antiretroviral therapy (HAART, or ART) among HIV patients in industrialised countries has been explored by a number of mathematical modelling studies [1-5]. The consequences of ART use are far from intuitive. Successful ART decreases plasma [6] and seminal viral load [7,8] and so is thought to reduce HIV infectiousness. However, its main function is to increase the life expectancy of infected individuals [9,10], and over time this causes the pool of potential transmitters of infection to grow. These two factors – decreased infectivity but increased duration of infectiousness – have opposing effects on transmission. In addition, increases in risk behaviour could result from increased optimism about HIV prognosis due to the availability of ART. This is an area of uncertainty, with contradictory evidence [11-15].

Mathematical models can be used to address questions regarding the potential impact and effectiveness of various strategies. In terms of ART use, they can be used to investigate:

1) optimising the efficient use of ART;

2) the epidemiological consequences of ART and interaction with behavioural changes/interventions;

3) the likely course of drug resistance evolution

a. Within the individual;

b. Between individuals;

4) achievable levels of coverage and effectiveness;

5) the effective and efficient use of second line treatments; and

6) demographic/health care impact.

This review will briefly describe a range of models investigating the impact of ART use in various settings and evaluate the utility of these dynamic models.

### The range of ART models

Mathematical models examining the epidemiological impact of ART broadly fall into two categories; those incorporating HIV transmission dynamics, where incidence of new infections is dependent on HIV prevalence [1,2,4,5], and simpler linear models [16-18]. A summary of ART models is provided in Table 1. Aalen et al [17] constructed a model describing men who have sex with men (MSM) in England and Wales and the use of ART. This Markov multi-stage model represented stages of HIV infection based on CD4 count. The authors considered a variety of treatment scenarios, and incorporated asymptomatic and symptomatic individuals and the concept of eligibility for treatment, making the simulation of treatment uptake and its impact more realistic than previous work. Wood et al [16] constructed a health economic model to predict the future impact of low-level ART use in South Africa from 2000 to 2005. The authors modelled total drug cost, cost per life year gained and the proportion of per person healthcare expenditure required to finance ART in each scenario. The study involved a cost effectiveness analysis comparing the epidemiological impact of ART with other interventions such as prevention of mother-to-child transmission (PMTCT). Freedberg et al [18] also used stages of disease determined by CD4 count and predicted the incremental cost per quality-adjusted year of life gained by ART in the US. Wilson and Blower [19] used a spatial mathematical model to explore ART allocation strategies among health care facilities in the province of KwaZulu-Natal, South Africa, with an emphasis on maximising equity in access to treatment.

**Table 1:** Summary of existing ART models, by date of publication.

**Study**	**Model structure**	**Setting**	**Assumptions**	**Key outcomes (pertaining to ART) and comments**
Zaric et al 1998 [74]	Dynamic, difference equations	MSM and IDU, US cities/regions	Infection stratified into HIV and AIDS stages. Allows acquired and transmitted drug resistance. Rate of resistance evolution: 95% per year for non-adherents, 5% per year for adherents. Increased life expectancy due to ART: 1.5-fold for adherents, 1.2-fold for non-adherents, 1.2-fold for adherents infected with drug resistant HIV.	The most important factor affecting emergence of drug resistance is adherence to ART.

Aalen et al 1999 [17]	Linear	MSM, England and Wales, 1990s	Markov multi stage model (stage based on CD4 count). Explicit diagnosis and treatment. Estimated HIV incidence in the MSM population, which was fixed at 1200 cases/year for all but one scenario, where incidence was halved from 1995 onwards.	Decrease in AIDS incidence due to ART. Number receiving treatment will increase 50–100% by 2001 compared to pre-1996.

Wood et al 2000 [75]	Linear model	IDU, Vancouver, Canada 1999–2006	Treatment uptake: 80% (scenario 1), 20% (scenario 2). Median increase in life expectancy due to ART: 7 years. No drug resistance or stratification by infection stage or sexual activity class. Prevalence estimates used in DemProj, part of the Spectrum suite of models (information at ) (3 scenarios: prevalence reduced from 7% in 1999 to 5% in 2006, prevalence remains at 7%, prevalence increases to 9% in 2006.	Calculated life expectancy and AIDS deaths 1999–2006 for each scenario. Concluded that low level ART use is not sufficient to increase life expectancy in this population and called for expansion in ART coverage.

Wood et al 2000 [16]	Linear health economic model (based on previous model [75])	South Africa 2000–2005	Median increase in life expectancy due to ART: 6 years (range: 5–7). Treatment uptake: 25% of infected adults. Spectrum AIDS Impact model was used to adjust the population projections for current and projected HIV-associated mortality.	Providing ART for 25% of the infected population could prevent a 3.1 year decline in life expectancy and more than 430,000 incident cases, but with disproportionate expenditure ($19 billion at 2000 prices) compared to preventing mother-to-child transmission.

Blower et al 2000 [5]	Dynamic, deterministic	MSM, San Francisco, US, up to 2010	Changes in sexual behaviour: no change to doubling of risk. Treatment coverage rates: 50–90% uptake per year. ART reduces infectivity 2- to 100-fold. Acquired drug resistance: 10–60% per year (infections can revert back to ART-sensitive). Resistance is transmitted, but is less fit than wild type. No stratification by stage of infection or sexual activity class. Increased life expectancy due to ART: 1.5- to 3.0-fold.	Increasing ART usage would decrease the death rate and substantially reduce HIV incidence (wide range of results due to uncertainty in parameter estimations).

Blower et al 2001 [63]	Dynamic, deterministic (used previous model [5])	MSM, San Francisco, US, 1996–2005	Treatment uptake and drug resistance evolution rates as for [5]. No change in risk behaviour. Assumed no resistant strains could arise that were as transmissible as wild type. Transmissibility range: 1–90% as fit as wild type. Implicitly allows superinfection with wild type virus of subjects with primary resistance.	Prevalence of ART resistance is already high in San Francisco and will continue to increase substantially through 2005. Transmitted drug resistance will remain low, only increasing gradually, with a doubling time of around 4 years and a predicted median 15.6% (range 0.05–73.21%) new infections resistant to ARVs by 2005.

Freedberg et al 2001 [18]	Linear health economic model	US	Monte Carlo simulation of a hypothetical cohort of infected patients. Disease progression predicted by CD4 count (6 categories) and viral load (5 categories). Detailed description and associated costs of HIV-related morbidity, opportunistic infections and death. Virologic failure represented as 0.5 log increase in viral load for 2 consecutive months. Increased life expectancy due to ART: 2 years.	The cost-effectiveness ratio for ART was $13,000–$23,000 per quality-adjusted life year gained. Initial CD4 count and drug costs were the most important determinants of costs, clinical benefits, and cost effectiveness.

Tchetgen et al 2001 [76]	Dynamic, deterministic	MSM (assumes same population as Blower et al [5])	No stratification by sexual activity or stage of infection (model is for all stages except AIDS; progression to AIDS exits an individual from the model population). Models diagnosis separately from treatment initiation. No sexual behaviour change due to ART. Drug resistance emerges at 1.2–13.5% per year for adherent patients and 67.3–85.9% per year for non-adherent patients. Resistant strains are half as transmissible as wild type. Untreated resistant infections may revert to wild type infections (10% per year). 60% of treated patients adhere. Increased life expectancy due to ART: approximately 3-fold. ART reduces infectivity by 74%. Withdrawal rates also vary by adherence.	Although screening for adherence is likely to reduce levels of drug resistance compared to treating all patients, HIV and AIDS incidence rates are likely to increase unless screening accuracy is extremely high.

Dangerfield et al 2001 [77]	Dynamic, deterministic	MSM, UK 1981–1998	Five stages of infection with varying infectivity broadly corresponding to primary infection, incubation, pre-AIDS and early and late AIDS. Three levels of sexual activity with proportionate mixing. No drug resistance. Proportion initiating ART at each stage (models 1,2 and 3 respectively): incubation = 0%, 0%, 60%; preAIDS = 0%, 10%, 25%; early stage AIDS = 0%, 10%, 35%. No uptake for late stage AIDS, which is defined as the final few months of care – authors assume patients only reach this stage as a result of treatment failure. Infectivity decreases to a constant level for all those treated, which is 35–40-fold less than for pre-AIDS.	Three models were designed, differing by prognosis of patients experiencing treatment failure for models 1 and 2. Model 3 stratifies life expectancy on ART by stage of infection at which treatment is initiated.

Law et al 2001 [4]	Dynamic, deterministic	MSM, Australia 1996	Population-level changes in sexual behaviour: no change to doubling of risk. Decrease in infectivity due to ART: 10-fold (range: 100-fold to none). Proportion of individuals diagnosed and treated increases with progression of disease, as determined by CD4 count. HIV diagnosis modelled separately to ART initiation, median 2-fold decrease in risk behaviour upon diagnosis (range: 25–75% reduction). No stratification by sexual activity group. No incorporation of drug resistance. Stratified by stage of infection in terms of CD4 count (>500 cells/ml, 200–500, <200, AIDS). Proportion treated by disease stage: >500 = 35%, 200–500 = 52%, <200 = 72%, AIDS = 90%.	Changes in risk behaviour were linearly associated with increases in incidence, while decreases in infectivity were non-linearly associated with decreases in incidence. Decreases in infectivity of 2-, 5- and 10-fold would be counterbalanced (in terms of incidence) by increases in risk behaviour of 40, 60 and 70%, respectively.

Velasco-Hernandez et al 2002 [1]	Dynamic, deterministic (used previous model [5])	MSM, San Francisco, US,	Previous model [5] is used to derive an analytical expression for R_0_. Used assumptions as for the previous model. Changes in risk behaviour: 50% reduction to 100% increase (whole population). Relative fitness of resistant strains: 1% to "approximately as transmissible".	Median R_0 _= 0.90 if risky sex decreased, 1.0 if risky sex remained stable, and 1.16 if risky sex increased. R_0 _decreased as ART coverage increased. The probability of epidemic eradication is high (p = 0.85) if risky sex decreases (median 25% reduction), moderate (p = 0.5) if it remains stable, and low (p = 0.13) if it increases (median 50% increase). Concluded that ART can function as an effective HIV prevention tool, even with high levels of drug resistance and risky sex, and could eradicate a high prevalence (30%) HIV epidemic.

Law et al 2002 [78]	Dynamic, deterministic (extension of previous model [4])	MSM, Australia 1996	Incorporation of other sexually transmitted infections (STI), but not dynamically-assumed 100% increase in prevalence of STI among all MSM regardless of HIV status, due to increased risk behaviour as a result of ART introduction. STI infection increased HIV infectivity 3.5-fold (range: 2–5-fold).	Decreases in infectivity of 2-, 5- and 10-fold would be counterbalanced (in terms of incidence) by increases in risk behaviour of 30, 50 and 65%, respectively i.e. even more modest increases than in previous publication [4]. Even small increases in STI as a result of increased risk behaviour could have an important multiplicative effect increasing HIV incidence.

Johnson & Dorrington 2002 [79]	ASSA2000 Interventions Model (dynamic, deterministic spreadsheet model)	South Africa	Stages of infection: stages I to IV of the WHO clinical staging system, with decline in sexual activity at advanced stages. Includes voluntary counselling and testing (VCT) with a corresponding (though transient) decrease in risk behaviour for both infected and uninfected individuals. Reduction in viral load due to ART: 1.76 log_10_. Reduction in infectivity: 67% per log reduction in viral load. 4 sexual activity classes. Only AIDS patients qualify for treatment. Model assumes a phased roll-out achieving 90% coverage by 2006. First 6 months of ART: death rate = 8.2%, discontinuation rate = 9.1%. Thereafter: death rate = 5.8%/year, discontinuation rate = 5.8%. Resistance not explicitly modelled.	ART provision is highly effective at preventing new infections, through reduced infectivity and assumed impact of VCT, and the high coverage level. ART plus VCT reduces incidence of AIDS, but because of increasing numbers starting treatment, the overall number of AIDS cases increases to a peak in 2015. Approximately one million deaths would be averted between 2001 and 2015 if ART is added to a set of AIDS prevention initiatives.

Nagelkerke et al 2002 [80]	Dynamic, deterministic	Botswana and India	Stratified by gender and 2 sexual activity groups (higher group represents CSWs and their clients). No changes in sexual behaviour due to ART, but "effective counselling" of those on ART could decrease infectivity of those developing drug resistance by 50%. ART reduces infectivity to zero. Rate of acquired resistance: 25% per year (range: 5–25%). Transmitted resistance possible (resistant strains appear to be as transmissible as wild type). Rate of treatment uptake: all those infected are recruited at rate 50% per year. No stratification by stage of infection.	Compared impacts of an ART programmes to other HIV interventions. Concluded that after transient success, ART would be ineffective within 30 years due to widespread drug resistance. Assumes high treatment uptake rates and pessimistic assumptions regarding transmission of drug resistant strains.

Gray et al 2003 [2]	Dynamic, stochastic	Rakai, Uganda, 2000–2020	Assumes ART reduces HIV log viral load by 27.0–42.5%, representing decreases in log viral load from 5.32 to 3.06 log_10 _copies/ml [49], and 5.23 to 3.82 log_10 _copies/ml [48]. These generate an average decrease in infectivity of 95.7% (0.0023 to 0.0001 per coital act) and 43.5% (0.0023 to 0.0013) respectively. Sexual activity decreases with increasing viral load. Behavioural disinhibition: increased risk by 50–100% (among those on ART only). Treatment uptake: scenario 1: all with viral load >55,000 copies/ml; scenario 2: all subjects, irrespective of viral load (20% of infected persons in Rakai had viral loads >55,000 copies/ml [81]). Range of treatment coverage: 0–100%.	Concluded that ART alone cannot control mature HIV epidemics such as that in Rakai. Behavioural disinhibition would counter decreases in HIV infectivity due to ART.

Xiridou et al 2003 [82]	Dynamic, deterministic	Young MSM, Amsterdam, The Netherlands	Steady and casual partnerships. Stages of infection: primary, incubation, AIDS. Sexual activity assumed to cease after development of AIDS. 42% subjects in incubation stage are diagnosed. Diagnosis during incubation results in a 25% (0–50%) reduction in risky behaviour. ART reduces infectivity by 74.5%. Increased life expectancy (before development of AIDS) due to ART: 9.5 years.	A 75–99% reduction in infectivity due to ART will be counterbalanced by increases of 50% (range: 30–80%) in risky behaviour with steady partners, but not by increases of up to 100% with casual partners. Increasing HIV testing from 42% to 80% and ART coverage from 70% to 85%, would mean even a 100% increase in risk taking with steady partners would not outweigh the effect of ART on HIV incidence.

Xiridou et al 2004 [3]	Dynamic, deterministic (extension of previous model [82])	Young MSM, Amsterdam, The Netherlands	Extension of original model [3] to allow for initiation of ART during primary infection. Proportion of men diagnosed during incubation and successfully treated: 60–80%. Proportion initiating ART during primary infection: 1–10%. ART reduces infectivity during incubation by 50–99%. Population-level increase in risk behaviour for both steady and casual partnerships: 0–100%. Mean incubation time to AIDS for initiating ART during incubation: 15–30 years. Decreases in infectivity and increases in life expectancy for those initiating ART during primary infection were forced to be larger than for those initiating ART during incubation.	Investigates the role of primary infection in HIV transmission. Estimates that among all new infections only 11% occur during primary infection. The effect of ART during primary infection on transmission is therefore limited. However, in a community with higher risk behaviour among casual partnerships, the fraction of transmission attributed to primary infection increases to 25%.

Clements et al 2004 [38]	Dynamic, deterministic (based on previous models [4, 78])	MSM, Australia 1995–2006	Assumed a 10% annual increase in population-level risk behaviour from 1996. A stable proportion receive ART from 1998, which then declines from 70% in 2001 to a median of 50% of diagnosed on ART by 2006.	HIV incidence was predicted to have declined during 1996–1998 due to ART, with a slow increase 1998–2001 due to increased risk behaviour while ART usage remained fairly stable. From 2001, a continued increase in risk behaviour coupled with a moderate decline in ART use would lead to a 50% increase in incidence by 2006.

Auvert et al 2004 [26]	Linear	Township near Johannesburg South Africa, 2002	Used results from cross-sectional study. Under WHO guidelines, all with CD4 counts <200 initiated treatment. Under USDHHS guidelines, all with CD4 counts <350 or viral load >55,000 copies/mL initiated treatment. Reduction in infectivity due to ART calculated using infectivity estimates by viral load category used by Gray et al [2] and comparing change in distribution of viral load in the community with and without ART.	Investigated short term impact of ART on incidence. The proportion of infected subjects eligible for ART was 9.5% (95% CI 6.1–14.9%) under WHO guidelines and 56.3% (95% CI 49.1–63.2%) under USDHHS guidelines. The population impact of ART on HIV transmission is small (reduction in annual risk of transmission 11.9% (95% CI 7.1–17.0%)) under WHO guidelines, but higher under USDHHS guidelines (71.8% (95% CI 64.5–77.5%))

Boily et al 2004 [30]	Dynamic, deterministic	MSM population	STI (gonorrhoea) increasing HIV infectiousness is modelled dynamically. Stratified into 6 sexual activity groups with proportionate mixing. Two stages of HIV infection: incubation and AIDS. AIDS patients treated with ART resume the sexual activity of asymptomatic individuals within their activity class. ART reduces infectivity by 25% (pessimistic), 50–90% (moderate), 99% (optimistic). Treatment uptake rates: 10–90% per year, for AIDS patients only, or for all infected subjects Withdrawal rate (due to treatment failure, resistance and toxicity): 0–50% per year.	Zero to 55% new bacterial STI could be attributed to widescale ART use, due to more modest increases in risky behaviour (0–25%) at the population level. These increases have a negative impact on HIV if coverage is too low. Increasing ART coverage helps to prevent more HIV infections despite larger increases in risk behaviour and STI that is predicted to ensue. No individual-level increase in risk behaviour; population-level increases in risk behaviour over time are due to ART slowing the depletion of high-risk infected individuals, so these populations are replenished.

Salomon et al 2005 [22]	The Goals model (linear spreadsheet model) (based on previous models [20, 21])	Sub-Saharan Africa, calibrated to 3 regions: East, West/Central and Southern, up to 2020.	Goals model adjusts UNAIDS/WHO EPP (epidemic projection package) and Spectrum model incidence and prevalence estimates. 5 different risk groups (single and married men and women, and CSWs). Median increase in life expectancy due to ART: 3 years. No drug resistance. Includes STI transmission. 3 stages of infection: primary, incubation and symptomatic. ART reduces infectivity by 99% (optimistic) or 66% (pessimistic). Number of partners reduced by 50% plus 2 times higher condom use (optimistic) or no change (pessimistic) for those treated. Risk behaviour of the general population does not change (optimistic), or condom use declines by 10% (pessimistic). Treatment uptake: 50% ART coverage (of those in need) by 2005, increasing to and remaining at 80% from 2010–2020. This "treatment-centred" response, where little prevention activity occurs, was compared to a "prevention-centred" response where no ART scale-up occurred, and a "combined response", with optimistic and pessimistic assumptions of the effect of ART on prevention efforts being investigated.	Explored the potential impact of ART in the context of a broader strategy for HIV/AIDS control, comparing deaths and new infections averted to baseline projections without interventions. A prevention-centred strategy provides greater reductions in incidence and mortality reductions similar to those of treatment-centred strategies by 2020, but more modest mortality benefits over the next 5–10 years. If treatment scale-up leads to reduced effectiveness of prevention efforts, benefits (in terms of infections and HIV/AIDS deaths averted) are considerably smaller than for initiatives which complement each other. The number receiving ART in 2020 ranges from 9.2 million in a pessimistic treatment-only scenario, to 4.2 million in a combined response scenario with positive treatment-prevention synergies.

Wilson & Blower 2005 [19]	Spatial model	KwaZulu-Natal, South Africa	Incorporates heterogeneity in treatment accessibility with distance to health care facilities, and heterogeneous distribution of people infected with HIV.	Determining the optimal ART allocation strategy among health care facilities, aiming to maximise equity. Authors' strategy gave more equal access to ART than allocating therapy to the state capital only, or equal allocation to all health care facilities.

In an investigation into the impact of an expanded response (incorporating prevention interventions and care and support activities) on the HIV/AIDS pandemic, Stover et al 2002 did not include the effect of ART because, "there is little empirical data available on the magnitude of the preventive effect of treatment (reduced viral load and hence infectiousness) and care" [20]. However in a later publication, the authors investigated the effects of combining treatment with effective prevention efforts, using the same model (the Goals model [21]), calibrated to sub-Saharan Africa [22]. The Goals model is a Microsoft Excel™ spreadsheet model using linear equations, designed to improve resource allocation for national HIV/AIDS programmes. It feeds into the dynamic epidemic projection package (EPP) and Spectrum, used by the UNAIDS/WHO to produce national HIV/AIDS estimates [23,24], to predict the impact of an intervention. The authors concluded that a prevention-centred strategy provides greater reductions in incidence, but more modest mortality benefits, than treatment-centred scenarios. A combined approach would yield further benefits, but focusing on treatment at the expense of prevention could diminish this effect.

Auvert et al 2004 used a linear model to estimate the proportion of the South African population requiring ART under the then current WHO guidelines (treating all individuals with a CD4 cell count less than 200 cells/mm^3 ^[25]) and to predict the impact of ART on the short term spread of HIV in this setting [26].

Such linear models have generally been used to inform policy makers on issues such as resource allocation, and typically involve only short-term predictions of the effect of ART for health care providers, as estimated by cost-effectiveness analysis [16,18]. The models are relatively straightforward in that they look at the health states of individuals, associated treatments and events that individuals experience, but fail to take account of the non-linear feedback process underlying infectious disease epidemics. Linear models are limited by the accuracy of estimates of HIV incidence used to parameterise the models, which is all the more important because their predictions and conclusions are usually more quantitative in nature than those provided by dynamic models, which have tended to be used to give more qualitative insight. Models incorporating HIV transmission dynamics typically investigate the impact of ART over a longer time frame and are used to address more general questions surrounding ART use, such as whether the benefits of ART provision outweigh the problems and risks, and which approaches to ART provision are most effective. Both types of model are required, to inform policy makers in resource-poor settings about the costs of ART provision in the short term (Wood et al [16], for example), as well as to predict the likely impact of scaling up ART use.

To date, policies designed to ameliorate the HIV/AIDS epidemic in Africa have been heavily based on policies from industrialised countries [27]. However, the epidemiological and economic contexts are so different that there is an urgent requirement to assess whether existing policy options and targets are optimal for resource-poor settings.

### Dynamic model structures

**Figure 1:**
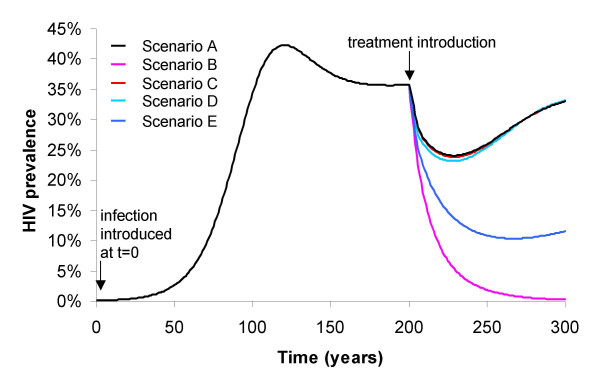
**Model predictions of the effect of ART on a mature epidemic, under various assumptions**. Model simulations of the potential impact of ART on a mature epidemic, varied by treatment uptake rate, reduction in infectivity due to treatment and impact on risk behaviour at the population level (see Endnote for model description). The model used only incorporates one stage of HIV infection and so individuals initiate treatment at an earlier stage of infection than is realistic, and there is homogeneous sexual mixing. Scenario A – ART uptake = 50% per year, sexual activity post ART = unchanged (2.5 partners per year), reduction in infectivity due to ART = 50-fold. Scenario B – ART uptake = 50% per year, sexual activity post ART halves (1.25 partners per year), reduction in infectivity due to ART = 50-fold. Scenario C – ART uptake = 50% per year, sexual activity post ART = unchanged (2.5 partners per year), reduction in infectivity due to ART = 1000-fold. Scenario D – ART uptake = 90% per year, sexual activity post ART = unchanged (2.5 partners per year), reduction in infectivity due to ART = 1000-fold. Scenario E – ART uptake = 90% per year, sexual activity post ART = reduced by 20% (2 partners per year), reduction in infectivity due to ART = 1000-fold.

Most dynamic models of HIV transmission investigating the impact of ART are deterministic, with a frequency-dependent (density-independent) transmission term. This means that the rate of (sexual) contact between one individual and others within a population does not depend on the density of the population, as it would, for example, in the case of contacts for air-borne infection transmission. HIV transmission models often incorporate relatively complex patterns of sexual behaviour, with model populations stratified into sexual activity groups by rate of partner change, and assuming different degrees of mixing between groups. However, to date most models specifically designed to examine ART impact have assumed homogeneous risk behaviour (although some of these models have investigated changes in risk behaviour of the general population as a result of ART introduction and/or a change upon diagnosis of HIV [4,5]). More realistic incorporation of sexual behaviour is likely to improve the ability of models to capture the observed timescale of African HIV epidemics, namely steady state being reached over decades rather than centuries. Figure 1 shows projections from a homogeneous sexual activity model, illustrating how, with a homogeneous population, realistic prevalence levels (representing epidemics in sub-Saharan Africa) can only be reached over unrealistic timescales (a full description of the model is provided in the Endnote). However, such homogeneous models can simulate HIV epidemics over realistic timescales if they are assumed to represent the 'at-risk proportion' of the total population only. This means that the population is crudely divided into two groups; one group practices no risky behaviour at all, whereas the other has a relatively high rate of (unprotected) sexual partner change. This structure produces an epidemic curve over a realistic timeframe (decades rather than centuries), without producing unreasonably high prevalence levels for the entire population (at-risk and not at-risk).

More sophisticated models incorporating sexual behaviour include partner models [28,29] and network models [30,31]. Gray et al [2] use a stochastic simulation incorporating individuals and their contacts, although some assumptions are not clear in the available publication. The need for complexity will depend on the nature of the research question [32]. For example, where changes in sexual behaviour as a result of ART are to be investigated, a more sophisticated description of sexual behaviour is required [30]. Where the effect of ART on transmission is to be investigated, a more realistic pattern of infectivity is required [2,4]. However, while increased complexity can make models more realistic, it also makes them more difficult to parameterise and it more difficult to analyse and interpret model output.

### Behaviour change

The possibility of widescale use of ART leading to changes in patterns of risk behaviour, particularly a disinhibition effect, has been of considerable concern. There are competing possible effects; at the individual level, treated patients may increase the frequency of sexual activity due to the severity of their symptoms decreasing, but may receive effective prevention counselling upon treatment initiation, which would decrease the frequency of risky activities. At the population level, in areas with substantial treatment coverage and successful treatment outcomes, there may be an increase in complacency among the general population regarding an HIV diagnosis, leading to increases in risk behaviour. Despite considerable debate [11-15], this relationship has not been convincingly demonstrated in industrialised countries where ART is readily available. A recent paper suggests that recent increases in risk-taking behaviour among MSM may be the result of non-volitional changes at the individual level over time [33]. The depletion of the pool of high-risk individuals in the pre-ART era made it more difficult for the remaining high risk-taking individuals to find partners to engage in risky sex with, but ART has facilitated the differential replenishment of this group. Therefore individuals who previously had to reduce their levels of risky sex could resume their initial high-risk behaviours.

The threat of behavioural disinhibition it is unlikely to be an immediate concern as ART is rolled out in high prevalence, resource-poor settings, where initial coverage is likely to be low and the effectiveness of ART programmes remains to be seen. Furthermore, the behavioural effects resulting from ART use in resource-poor settings are unlikely to follow patterns of industrialised countries. A person's decision to have sex, protected or unprotected, is influenced by a different set of considerations in resource-poor settings than those common in industrialised countries. Key is an individual's ability to negotiate her or his own sexual activity – as defined by fear of stigma, financial need, or the status of women within society. An individual is also less likely to be aware of his or her serostatus, due to lack of testing facilities and/or fears regarding a positive result. The provision of treatment may increase interest in voluntary counselling and testing (VCT) services, which may in turn lead to a decrease in frequency of risk behaviour by those infected. In Cote d'Ivoire for example, individuals reported low sexual activity following an HIV diagnosis, and this was not increased by the offer of ART [34]. Despite the inaccuracies of sexual behaviour data, these results are encouraging.

Given that it is difficult to predict how individuals might change their sexual behaviour as a result of ART introduction in different regions, models are faced with either estimating behavioural parameters from epidemiological data, or exploring pessimistic and optimistic scenarios using parameter values assumed to be at the ends of the spectrum of possible outcomes. Law et al 2001 modelled the effect of ART on the HIV epidemic in Australia in 1996 among the homosexual population [4] and predicted the outcome of the competing effects of increased life expectancy, decreased infectiousness and increases in unsafe sex of uninfected MSM on HIV incidence. Their assumption of a range of no change to a doubling of risky sex was essentially arbitrary, but demonstrated that increases in sexual behaviour (and life expectancy) could negate the beneficial impact of decreased infectiousness on incidence. Blower et al 2000 produced similar results for the homosexual population in San Francisco [5], again using the range of no change to a doubling in sexual risk-taking.

Velasco-Hernandez et al have investigated the conditions under which ART in HIV infected individuals may drive an epidemic to extinction [1]. As can be shown by the model output in Figure 1, for ART to eliminate HIV, an extensive reduction in risk activity at the population level, accompanying ART use (such as a 50% reduction in the partner acquisition rate) is required, together with high levels of treatment uptake and large decreases in infectiousness induced by ART. As behaviour change is notoriously difficult to generate and initial coverage rates for ART in resource-poor settings are likely to be low, this optimistic scenario is highly unlikely.

The early impact of widescale ART use in resource-poor settings where HIV prevalence is currently high will probably not involve substantial population-level increases in risky behaviour. The effectiveness of local ART programmes will likely have to be demonstrated across a broad swath of the population before the perceived threat of AIDS as a disease declines. In lower prevalence regions where high coverage rates are feasible, such changes may occur. Careful monitoring of potential changes in risk behaviour would be very useful, if feasible. Any model designed to explore the impact of sexual behaviour change in resource-poor settings, be it an increase or a decrease, should explicitly model HIV diagnosis separately from treatment initiation, as shown by Law et al [4], because 1) it is knowledge of HIV status and the associated counselling that may change behaviour, 2) the advent of therapy in the sick may change their desire and/or ability for sexual functioning and 3) the attitude of those who know they are infected with HIV may change between not being treated, where they perceive a risk of transmitting to partners, to being treated, where the magnitude of risk may be perceived as smaller. This is one area where the introduction of ART could be used for prevention as well as treatment, through facilitating VCT.

### Stage of HIV infection

Some researchers believe that ART could be used as a direct prevention tool due to its effect on viral load leading to a decrease in infectivity and therefore incidence [26,35,36]. However, the competing effects of increasing prevalence due to the effect of ART on life expectancy and potential behavioural disinhibition would make this a risky strategy. Furthermore, models that predict dramatic reductions in incidence due to ART have used unrealistic treatment uptake rates. As described, some have argued that even high prevalence (30%) epidemics can be driven to extinction by ART, when assuming a treatment coverage rate of 50% to 90% [1,5]. The 50% level was estimated from data collected in a telephone sample interview of 462 MSM from four US cities conducted between November 1996 and February 1998 [37]. This was when HAART was in its infancy and treatment was initiated in a large proportion of HIV positive individuals, regardless of infection stage or CD4 count, because a "hit hard, hit early" consensus existed for patient management. Furthermore, the study only included self-identified, HIV-positive MSM, and so individuals unaware of or reluctant to admit their serostatus would have been missed. It is now more common to initiate treatment at a later stage of infection, due to side-effects and the risk of evolution of drug resistance. The proportion of HIV-infected people currently being treated, even in industrialised countries, is likely to be substantially below that required for any prospect of disease elimination.

More realistic patterns of ART use are incorporated in the models of Law et al [4,38] and Gray et al [2], where the proportion of individuals treated increases with severity of HIV disease as determined by CD4 count [4] or plasma viral load [2]. By explicitly modelling changes in infectiousness and sexual activity over time, it has been shown that ART alone cannot be relied upon as a sole prevention tool.

Gray et al [2] and Nagelkerke et al [39] explicitly modelled the impact of ART in resource-poor settings (Uganda, and Botswana and India, respectively). Nagelkerke et al 2002 assumed that those receiving ART and infected with drug-sensitive virus had zero infectivity, which does not reflect the true situation, despite viral load being substantially reduced [39]. Assumed rates of resistance evolution seem optimistically low for ART use in resource-poor settings, only being varied between 5% and 25% of those on ART failing treatment per year, whereas rates as high as 60% have been predicted by others [40-42]. Despite this the model predicted that after transient success, ART would be rendered ineffective within 30 years due to wide-scale emergence of drug resistance, based on resistant virus being as transmissible as sensitive virus.

Gray et al's conclusions were relatively pessimistic [2], contrasting with Blower et al [5]. Gray et al concluded that ART alone cannot control mature HIV epidemics such as that in Rakai, Uganda. This conclusion concurs with Garnett et al 2002 [43], who believe that ART cannot make an impact on a mature epidemic unless treatment is initiated with high coverage and earlier in infection (i.e. with higher CD4 cell counts) than is currently recommended in treatment guidelines. Such early treatment is unfeasible financially and unwarranted clinically, since it would lead to earlier evolution of resistance and treatment failure, leaving individuals running out of treatment options, perhaps even before the onset of AIDS.

The dependence of the epidemiological impact of ART use on the timing of treatment initiation is worth considering in more detail. The progress of HIV infection to AIDS can broadly be divided into four stages: primary infection, incubation, the period preceding AIDS ("pre-AIDS") and AIDS. While there is much between- and within-patient variation, on average, infectiousness is highest during primary infection, pre-AIDS and AIDS. Some experts believe that primary infection carries the highest risk of transmission, because it is associated with high plasma HIV RNA levels and continued sexual activity [44]. However, while some studies are aiming to evaluate the effect of treating individuals in primary infection [3], the vast majority of HIV infections are not diagnosed until well into the incubation period. If primary infection is defined as the period before detectable antibodies against the virus emerge, then testing can only identify those who have completed the primary stage. However, if primary infection is used to describe the high initial viraemia then infection could be diagnosed before this has ended. Treatment could not start earlier than the incubation stage which follows primary infection except in rare circumstances where exposure is known to have occurred. In resource-poor settings, diagnosis is frequently at a very late stage of infection [45,46], partly because of the non-specific nature of symptoms and the difficulty in accessing healthcare. Therefore, initiating treatment at diagnosis or when CD4 counts descend to a certain benchmark, such as 350 or 200 cells/mm^3 ^(as recommended by current guidelines [25,47]), will mean that the highly infectious period of primary infection and the long period of incubation escape the controlling effects of treatment.

**Figure 2:**
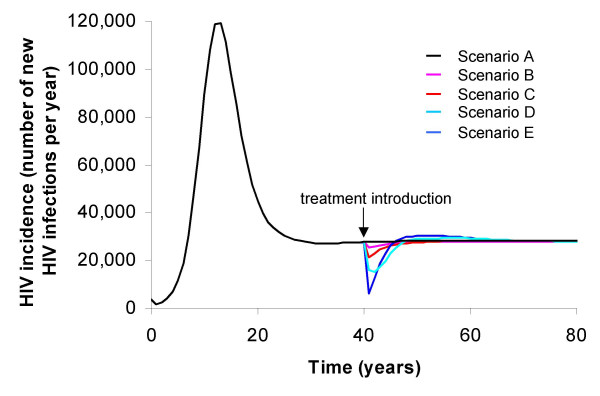
**Predictions of the impact of ART by stage of infection at which treatment is initiated**. Predictions of the impact of the introduction of ART in terms of HIV incidence, by stage of infection at which treatment is initiated (for a brief description of the four stage infection model used, see Endnote). Scenario A – No treatment. Scenario B – ART uptake: AIDS patients only (after a mean of 1 month). Scenario C – ART uptake: AIDS patients (after mean 1 month) and pre-AIDS (after mean 6 months). Scenario D – ART uptake: AIDS patients (after mean 1 month) and pre-AIDS (after mean 6 months) and incubation stage (after mean 4 years). Scenario E – ART uptake: all four stages, after mean 1 month.

In models examining the impact of ART on HIV incidence, inclusion of the variation in infectiousness as a function of infection stage is crucial for producing realistic predictions. As ART can only be initiated upon HIV diagnosis, it will have no effect on transmission from most individuals undergoing primary infection, when risk of transmission is high. By the time an individual has developed AIDS, their sexual activity will have decreased, and so this group of infected individuals will not contribute as much to HIV transmission as the duration of this phase would suggest. Figure 2 shows runs from a four-stage HIV infection model, with various treatment coverage scenarios, determined by stage of infection. Treatment is introduced into a population with a mature HIV epidemic and a high basic reproductive number for the at-risk fraction of the population (R_0_~5), so it is not surprising that even aggressive implementation of ART to individuals, regardless of stage of infection, cannot lead to elimination. Figure 2 illustrates that ART under more realistic assumptions regarding treatment delivery, in terms of treatment initiation, will have far less impact on incidence. In this model, there is a single treatment regimen and high, but plausible, rates of drug resistance evolution (30% per year), meaning that the effects on transmission are short-lived, coinciding with the effectiveness of the regimen. This illustrates the urgent need for cheap and reliable second-line treatment options to be available for ART roll-out.

Despite our view that Blower et al are over-optimistic [5], Gray et al's assumptions of the effects of ART may similarly be over-pessimistic [2]. The authors assume that ART leads to an average proportional reduction in HIV log viral load of between 26.8% and 43.6%, based on data from the Women's Interagency HIV Study (WIHS) [48] and the John Hopkins Clinic [49] respectively. However, other studies distinguish between patients who respond to a regimen (who typically experience reduction in viral load to undetectable levels (<50 copies/ml)), those who do not respond and those who subsequently experience treatment failure (viral rebound). With these distinctions, an individual responding successfully to ART will have a far greater reduction in viral load than Gray et al assume. Furthermore, the proportion reduction was the value recorded one month and three months after treatment initiation for the WIHS and John Hopkins Clinic patients, respectively. It can take much longer than this for complete reduction of viral load, often to undetectable limits [50] (models generally do not explicitly account for a delay between treatment initiation and effect, but it can be assumed that this is implicitly accounted for in the treatment uptake rate). Gray et al also included the possibility of behavioural disinhibition; the average number of partners for those on treatment was increased by 50% or 100%. Again, the values appear pessimistic and were essentially chosen arbitrarily, probably in order to complement other models [4,5].

### Emergence of ART drug resistance

Many models of ART have concentrated on predicting the emergence and spread of ART drug resistance, which has been of concern [51-54]. Once again it is very difficult to make such predictions, as the spread of drug-resistant virus is highly dependent on the replicative fitness of the resistant strains that evolve and their ability to superinfect individuals infected with wild-type strains (i.e. to co-infect someone already infected with wild-type virus, and successfully replicate). Superinfection is perhaps only likely in the successfully treated individual, where suppression of viral load allows the target cell population to recover, hence increasing the chance of successful replication and establishment of a new strain. In the untreated individual, it is unlikely that low frequency resistant virus, typically less fecund than wild type in this environment, would be able to compete against the established viral population sufficiently successfully to allow long-term persistence of the invading strain.

In a context where ART use is common in core groups, the possibility of superinfection of those on ART means that the likely maximum rate of spread of resistance epidemics may be similar to the speed of the initial HIV epidemic. HIV co-infection with different wild-type viruses [55,56], and by wild-type strains re-infecting patients harbouring drug-resistant viruses after a short period of treatment interruption [57,58], have both been documented. Chakraborty et al postulate that it is possible for patients infected with wild type HIV-1 isolates and under successful ART to become exposed to drug-resistant strains that would have significant selective advantage, leading them to outcompete the original wild-type strain and instigate treatment failure [59]. They concede that the probability of an individual undergoing successful treatment of a wild-type strain being exposed to a drug-resistant strain is low, but the large-scale roll-out of ART in high prevalence, resource-poor settings may increase this probability substantially.

The rate at which drug resistance evolves within the individual is likely to become higher in resource-poor settings than industrialised countries; even though there are reports of patient adherence being no lower than in the West [60], potential interruptions in supply due to transport problems and a lack of sophisticated laboratory monitoring systems will limit the success of any ART regimen. However, even with high levels of drug resistance evolving within the individual ("secondary resistance"), transmission of such strains ("primary resistance"), while increasing in many industrialised settings [54,61], is reported to be substantially less frequent than for wild-type HIV [61,62], because there is usually a fitness cost for mutations. Mathematical models may have the ability to predict best- and worst-case scenarios for resistance spread [39,63], but it must be conceded that the degree to which drug resistance and risky behaviour increase as ART use rolls out in Africa and other resource-poor areas cannot yet be quantified.

Blower et al 2001 predicted that acquired resistance will continue to rise, but transmitted resistance is likely to increase only gradually, with a doubling time of around four years and a predicted median of 15.6% of new HIV infections likely to be resistant to antiretroviral drugs by 2005 [63]. This conclusion was due to an assumption that of all possible ART-resistant HIV strains that could possibly evolve, none could be as transmissible as wild-type. The study also assumed that individuals infected with ART-sensitive virus undergoing treatment cannot be co-infected or superinfected by an ART-resistant strain.

Despite the conclusion that transmitted ART resistance will stabilise at low levels, the predicted range around the 15.6% value is very wide (0.05% to 73.21%) [63]. The authors argue that the higher values in the range generated from their sensitivity analysis have a very low probability. However, the choice of parameter distributions in the Monte Carlo sampling of parameter space undertaken in their study was arbitrary (in the sense of not being motivated by prior data) and entirely determines the probability of pessimistic scenarios.

The authors themselves acknowledge that they are "predicting the unpredictable" [63], but argue that their theoretical predictions [5] are in close agreement with empirical data [64]. Both display an increase in primary resistance between 1997 and 2001, but this is a short time period and the increase may reflect the expansion in use of ART over this time. Furthermore, the uncertainty interval around predictions made by the authors is large enough for a wide range of empirical data to fit the model. Blower et al acknowledge that transmission of ART resistance may vary widely by location and that frequent comparison to empirical data is necessary. However, Blower et al in 2005 recommend that large-scale surveillance for detecting transmitted resistance in Africa will be unnecessary for the next decade because transmitted drug resistance will not reach more than 5% during that time [65]. This is due to the assumption that ART use will remain at low levels, although the authors suggest that in urban locations rates of treatment may be higher. They recommend close monitoring of treated patients, but in areas where resources are constrained, this is unlikely to be practicable (WHO guidelines do not consider resistance testing, or even viral load testing, to be a priority in these regions [25,66]). We would argue that surveillance of the prevalence of drug resistance among patients is required in all locations where ART is used, and that the predictive utility of models with high degrees of uncertainty in their input parameters, and hence also their results, is limited.

In this context, it should be noted that increases in levels of acquired resistance are not inevitable – in Switzerland, where more than 80% of prescribed ART is dispensed by one of the highly experienced Swiss HIV Cohort centres, prevalence of drug-resistant HIV in newly infected individuals has been decreasing since 1996 [67]. However, we would argue that such an effect is less likely in resource-poor settings with restricted access to high-quality care and laboratory facilities and potential problems of drug sharing, black market resale of drugs and inappropriate prescribing of mono and dual therapy outside of official ART programmes [53]. The differences between ART programmes in industrialised and developing countries will be so marked that predicting programme impact and patterns of drug resistance from those in former setting is not necessarily informative.

**Figure 3:**
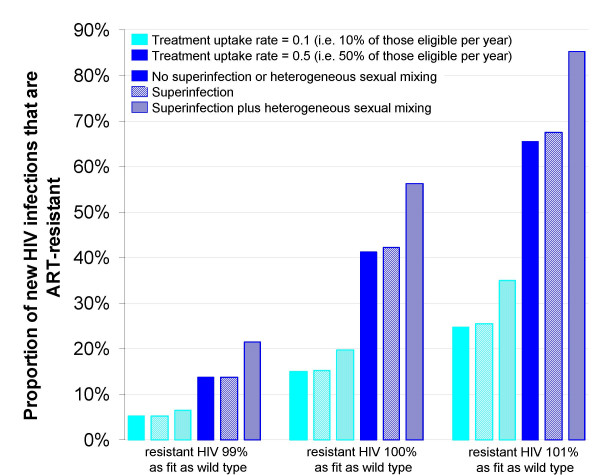
**Model predictions of transmission of ART drug resistance by relative fitness of strains**. Predictions of the spread of transmitted (primary) ART resistance under various scenarios, using a simplified ART model (see Endnote). Model output is 10 years after ART introduction. ART is introduced once the epidemic has reached equilibrium. Superinfection refers to the infection with ART-resistant HIV of individuals previously infected with ART-sensitive HIV and successfully undergoing treatment. These are the only individuals without viral outgrowth, and thus will have a pool of target cells rendering them susceptible to infection. Evolution of drug resistance within an individual is at a rate of 10% per year.

If one relaxes the assumption that no resistant strain can exceed the transmission fitness of wild-type even in the presence of ART use, even greater variation in predicted levels of transmitted drug resistance after 10 years of ART provision is possible (Figure 3). The scenarios illustrated assume a conservative rate of 10% per year for the evolution of resistance in the treated patient and do not allow for the possible enhancement of that rate in individuals suffering viral rebound (an increase in viral load following a previous decrease due to ART) without initial resistance, but who are then maintained on the same regimen (due to a lack of virological testing). Nevertheless, the results show that if a relatively fit variant emerged, the effectiveness of current ART regimens could be compromised after a very short period. There appears to be little change in model results when superinfection alone is allowed to occur, but when heterogeneous sexual activity is incorporated, resistance transmission is predicted to emerge more rapidly. This is due to superinfection allowing resistance to be transmitted through core groups receiving ART.

These results do not suggest a *likelihood *of drug resistance transmission but merely demonstrate the potential effects of various scenarios. It is more for biological studies (in particular resistance testing) and within-host models of HIV infection to examine the possibility that such strains could emerge [68-70]. Even in instances where laboratory tests reveal infections with virus deemed "resistant" to more than one drug class (either phenotypically or genotypically), these infections often still respond to treatment. A multidrug-resistant HIV strain would require a large number of compensatory mutations to be of a comparable fitness to wild type strains. However, ongoing treatment pressure in the presence of viral rebound could lead to sequential mutations increasing the fitness of a resistant strain; therefore, there is an argument for close monitoring of patients and implementation of drug resistance surveillance systems as ART is rolled out in resource-poor settings.

### Parameterisation

To aid the design of successful ART programmes in resource-poor settings, more information on the impact of ART provision is crucial – data on morbidity and mortality, tolerability, treatment failure and the possible emergence of drug resistant strains are very important. This information also increases the reliability of model predictions by providing more accurate ranges of parameter estimates. Population-level monitoring for changes in risk behaviour and patterns of ARV drug resistance are also required. Information on the performance of ART programmes in these settings is starting to be generated. Pilot programmes such as the Médecins Sans Frontières (MSF) initiatives (established in seven low- and middle-income countries: Malawi, Kenya, South Africa, Cameroon, Cambodia, Thailand and Guatemala) are starting to report back preliminary findings. The six-month outcomes from the MSF projects were positive [71]: the probability of survival at six months was estimated as 89.5% (95% CI 86.8–92.1), with high patient attendance and adherence rates comparable to those in industrialised countries. However, pilot programmes may not be representative of future large-scale ART roll-out, where health-care infrastructure and expertise are likely to be poorer. Other programmes, such as those of the Drug Access Initiative (DAI), formed in 1998 by UNAIDS in collaboration with the Ministries of Health of Chile, Côte d'Ivoire, Uganda and Vietnam, have been running for longer. Reports from the first two years of the initiatives in Côte d'Ivoire have been positive [72], with immunologic and virologic outcomes similar to those reported from industrialised countries. The only resource-poor country to implement ART provision on a large scale is Brazil, which has made ART available free of charge to all eligible patients since 1996, and has produced positive outcomes [73]. The experience of Brazil can give information on the impact of long-term, large-scale ART provision, but is a very different setting to sub-Saharan Africa. As with data from industrialised countries, care must be taken in interpreting and assessing the applicability of results. Modelling provides the tools to predict the consequences of possible activity; by constraining ourselves to examining only those scenarios that are supported by current, gathered data, we can be ignoring other, distinct possibilities, such as the case in which an ART-resistant strain as fit as wild-type could evolve.

## Conclusion

We have argued that HIV transmission models predicting the impact of ART use should incorporate a realistic progression through stages of HIV infection in order to realistically capture the timing of treatment initiation. Further elaboration of models is required (depending on the research question being posed), in areas such as time of diagnosis, sexual behaviour and assumptions regarding drug resistance evolution and transmission. All modelling studies are eventually dependent on the availability of setting-specific surveillance and behavioural data, and collection of such data is important for all regions where large scale ART use is introduced.

More investigation is required in order to determine the effect of introducing ART on a substantial scale in resource-poor settings with different stages and magnitudes of HIV epidemic. Models addressing questions of ART implementation in such settings, utilising data from fledgling ART projects where possible, will be of great use in designing cost effective programmes.

## List of abbreviations

AIDS Acquired Immunodeficiency Syndrome

ART Antiretroviral therapy

HAART Highly Active Antiretroviral Therapy

HIV Human Immunodeficiency Virus

MSM Men who have Sex with Men

PMTCT Prevention of Mother to Child Transmission

VCT Voluntary Counselling and Testing

## Endnote

### Model assuming one stage of HIV infection

**Figure 4:**
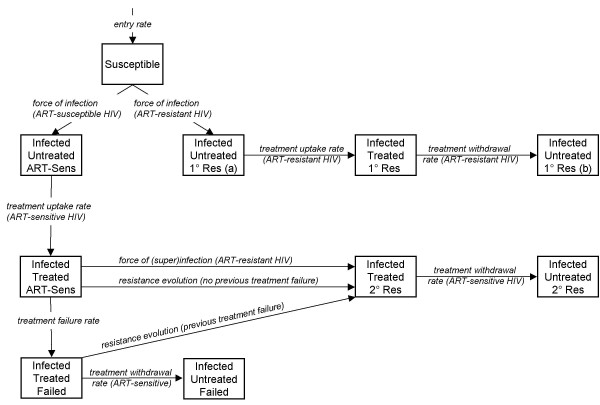
**Model of HIV transmission and treatment, with one stage of infection only**. Schematic illustration of the structure of the one stage HIV transmission model. 1° Res designates those with primary (transmitted) resistance, while 2° Res designates those with secondary (acquired) resistance. ART-Sens denotes people infected with ART-sensitive virus. For clarity, death rates are not shown.

The model assuming one-stage of HIV infection, used to produce Figure 1, is illustrated in Figure 4, with state variables, parameter symbols and model equations given below. Superinfection with an ART-resistant strain is possible for individuals undergoing successful treatment only (, S = ART-sensitive, T = treated), as these are the only individuals without viral outgrowth, and thus will have a pool of target cells rendering them susceptible to infection. Treatment failure can occur with (κ) or without (*f*) the evolution of drug resistance. Individuals who have developed treatment failure not accompanied by resistance (, F = treatment failure) are at increased risk of developing drug resistance (κ_*F*_) because of viral replication in the presence of continued drug pressure. Figure 3 uses a version of this one-stage model, modified to incorporate heterogeneous sexual mixing, with four different sexual activity groups.

The advantage of one-stage models of infection is that they are relatively simple and analytically tractable; that is, the relationship between each parameter and the outcome of the models, for example in terms of R_0_[1], can be exactly specified without recourse to simulation and sensitivity analysis. However, while a model should not incorporate complexity for its own sake, stages of HIV infection play a crucial role in the impact of ART, because treatment is only initiated at late stages of infection, and infectivity and sexual activity vary with the course of infection. Similarly, incorporating heterogeneous sexual activity within a model is more important for some research questions than for others. If we want to investigate the potential impact of a transmissible ARV-resistant HIV strain through a population, its spread would appear very different in a model of homogeneous sexual activity, compared to one with heterogeneity and various assumptions regarding mixing between activity classes, where infection would travel through core groups first before spreading into the general population.

### State Variables

*S *= susceptible individuals

 = ART-sensitive HIV infected individuals, untreated

 = ART-sensitive HIV infected individuals, treated

 = ART-sensitive HIV infected individuals, treated but viral rebound

 = ART-sensitive HIV infected individuals, suffered viral rebound, withdrawn from treatment

 = ART-resistant HIV infected individuals, treated

 = ART-resistant HIV infected individuals, untreated

 = ART-resistant HIV infected individuals (transmitted resistance), untreated

 = ART-resistant HIV infected individuals (transmitted resistance), treated

 = ART-resistant HIV infected individuals (transmitted resistance), withdrawn from treatment

λ_*S *_= force of infection for ART-sensitive virus phenotype

λ_*R *_= force of infection for ART-resistant virus phenotype (and force of (super)infection for ART-resistant virus phenotype, infecting an HIV wild type infected individual under treatment pressure)

### Parameters

For individuals of infection status :

*i *refers to viral phenotype (*S *(ART-sensitive) or *R *(ART-resistant));

*j *refers to treatment status (*U *(untreated) or *T *(treated)).

σ*N*_0 _entry into model population (rate of recruitment into sexually active class). *N*_0 _is the size of the population at time *t *= 0.

μ death rate due to causes other than HIV infection

 excess death rate due to HIV infection for an infected individual in class 

γ_*S *_treatment uptake rate for individuals infected with ART-sensitive HIV

γ_*R *_treatment uptake rate for individuals infected with ART-resistant HIV

*f *rate of treatment failure (viral rebound) without ART resistance evolution

κ rate of resistance evolution without previous viral rebound

κ_*F *_rate of resistance evolution with previous viral rebound

α_*S *_rate of treatment withdrawal for individuals initially infected with ART-sensitive HIV

α_*R *_rate of treatment withdrawal for individuals initially infected with ART-resistant HIV

 HIV transmission probability per partnership for an infected individual in class 

*c *Number of sexual partnerships per year

 Probability of  transmitting ART-resistant HIV

 Probability of  transmitting ART-resistant HIV

 Probability of  transmitting ART-resistant HIV

 Probability of  transmitting ART-resistant HIV

ι Factor reduction in transmissibility for an infected individual in class 

### Transmission equations



The forces of infection for ART-sensitive (λ_*S*_) and ART-resistant (λ_*R*_) HIV are given below. They are determined by the infectiousness of individuals in each class (β) and the probability that ART-resistant rather than ART-sensitive virus is transmitted in the case of mixed infections (ω).



### Model assuming four stages of HIV infection

**Figure 5:**
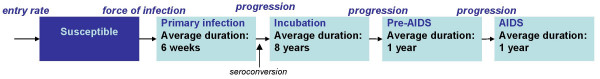
**Model of HIV transmission and treatment, with four stages of infection**. Schematic illustration of the structure of the stages of HIV infection for the four stage model. Individuals progress from one stage to the next exponentially, with an average duration in each stage as shown in the figure. Individuals have no HIV-related mortality during primary infection or incubation, a very slightly elevated baseline death rate during pre-AIDS and an average one year life expectancy once AIDS has developed.

The model used to produce Figure 2 is essentially the same as for the one stage infection model, but with infection divided into four stages (primary infection, incubation, pre-AIDS and AIDS) as shown in Figure 5. The forces of infection are determined by the infectiousness of individuals in each class, the probability that ART-resistant rather than ART-sensitive virus is transmitted in the case of mixed infections and the rates of sexual partner change, which decrease as individuals reach the final stage (AIDS). The rest of the model structure is as for the one-stage model. Full details of this model, equations and parameter estimates are not shown here but are available on request.

## Competing interests

RB was supported by an unrestricted education grant from GlaxoSmithKline.

## Authors' contributions

RB drafted the manuscript and constructed the mathematical models. All authors reviewed articles and read and approved the final manuscript.
